# Extensive antimicrobial resistance and plasmid-carrying resistance genes in mcr-1-positive *E. coli* sampled in swine, in Guangxi, South China

**DOI:** 10.1186/s12917-021-02758-4

**Published:** 2021-02-18

**Authors:** Jingzhi Yuan, Xiaoye Wang, Dali Shi, Qiang Ge, Xingxing Song, Wen Hu, Deyuan Wei, Chenling Ge, Xun Li, Chuanhuo Hu

**Affiliations:** College of Animal Science and Technology, Guangxi University, Nanning, 530004 P. R. China

**Keywords:** Swine-origin multi-drug resistance MCRPEC, Antimicrobial resistance, Extensively drug-resistant, Acquired antimicrobial resistance genes

## Abstract

**Background:**

The discovery of the superbug *mcr-1*-positive *Escherichia coli* (MCRPEC) has drew greet attention. Swine-origin multi-drug resistant MCRPEC has been a potential threat to public health and safety. However, there were few detailed studies have been reported on swine MCRPEC in Guangxi, South China.

**Results:**

In this study, thirty-three MCRPEC strains were detected from 142 *E. coli* strains from 116 samples in Guangxi in 2018. Which could be classified into eight unique STs and a total of six incompatibility plasmid groups (IncFI, IncHI1, IncY, IncN, IncI1 and IncX1). After that, the susceptibility of MCRPEC isolates to 27 antimicrobial agents belonging to 17 antimicrobial categories was tested. There were nineteen *E. coli* resistant to 3rd and 4th generation cephalosporins and twelve *E. coli* resistant to carbapenem resistan. Importantly, the MCRPEC showed high resistance highly resistance for imipenem and meropenem, which were forbidden to use in livestock production. Three MCRPEC strains were further proved to be extensively drug-resistant (XDR), and the other isolates were multi-drug-resistant (MDR). Furthermore, we found that the plasmid-carrying resistance genes coexisted with the *mcr-1* gene of the MCRPEC isolates. Which were listed as follows: β-lactamase antimicrobial resistance genes e.g. ESBL genes (*bla*_*CTX-M14*_, *bla*_*CTX-M24*_, *bla*_*CTX-M123*_, *bla*_*OXA-1*_), plasmid-mediated AmpC (pAmpC) gene (*bla*_*CMY-2*_), the carbapenem resistance gene (*bla*_*NDM-5*_), and non-β-lactamase antimicrobial resistance genes (*qnrA*, *qnrB*, *qnrS*, *aac (6′)-Ib-cr*, *tetA*, *tetB*, *sul1*, *sul2*, *floR*, *aadA*).

**Conclusion:**

Thirty-three *mcr-1*-positive *E. coli* isolates in Guangxi displayed a wide profile of antimicrobial resistance. Plasmid-carrying resistance genes might be the main cause of MCRPEC multidrug resistance. This study highlighted the necessity for long-term surveillance of *mcr-1*-positive *E. coli* in pigs.

**Supplementary Information:**

The online version contains supplementary material available at 10.1186/s12917-021-02758-4.

## Background

Superbug infections are one of the most serious threats to public and animal health nowadays. The emergence and rapid spread of multi-drug-resistant (MDR), extensively drug-resistant (XDR) and pan-drug-resistant (PDR) bacteria has been a major public health problem worldwide [1]. MDR was defined as acquired non-susceptibility to at least one agent in three or more antimicrobial categories, XDR was defined as non-susceptibility to at least one agent in all but two or fewer antimicrobial categories (i.e. bacterial isolates remain susceptible to only one or two categories) and PDR was defined as non-susceptibility to all agents in all antimicrobial categories [2]. The transmissibility of antimicrobial resistance mediated by mobile plasmids was reported to be an important reason for the generation of XDR and PDR bacteria [3].

In China, colistin is the last line of defense against carbapenem-resistant *Escherichia. coli* (CREC) [4, 5]. However, colistin has been used in animal production in China for decades as a treatment and feed additive [6]. In 2015, the plasmid-mediated *mcr-1* gene was first discovered in food animals in South China [7]. Subsequently, nine different *mcr* alleles, e.g. *mcr-1* to *mcr-9*, were found in different bacteria from many countries and regions [7–15].

Due to the extensive use of β-Lactam antimicrobial in human and veterinary medicine, the number of extended-spectrum β-lactamase (ESBL)-producing *E. coli* is increasing rapidly worldwide [16, 17]. More related studies on CREC were reported recently, for CREC is multi-drug resistant which is difficult to treat and cause a high lethality after infection [18, 19]. The occurrence of colistin resistant CREC aggravate the situation [20, 21].

Thus, this study aimed at investigate the phenotype of antimicrobial resistance of MCRPEC and its plasmid-carrying resistance genes from pigs in Guangxi, South China.

## Results

### Identification of *mcr-1* positive *E. coli* (MCRPEC) isolates

A total of 142 *E. coli* isolates were isolated from pigs with diarrhea/dyspnea in Guangxi in 2018. Seventy-two (50.7%, 72/142) *E. coli* isolates were tested with colistin (MIC 3.5 mg/L). To investigate the proportion of *mcr* genes in *E. coli*, PCR amplification was performed to test *mcr-1*, *mcr-2*, *mcr-3*, *mcr-4*, *mcr-5*, *mcr-6*, *mcr-7*, and *mcr-8*. 33 *mcr-1*-positive *E. coli* strains detected. The percentage of MCRPEC strains accounted for 45.8% (33/72) of colistin resistant strains and 23.2% (33/142) of all isolated strains. The thirty-three MCRPEC isolates were used for subsequent study.

**Fig. 1:**
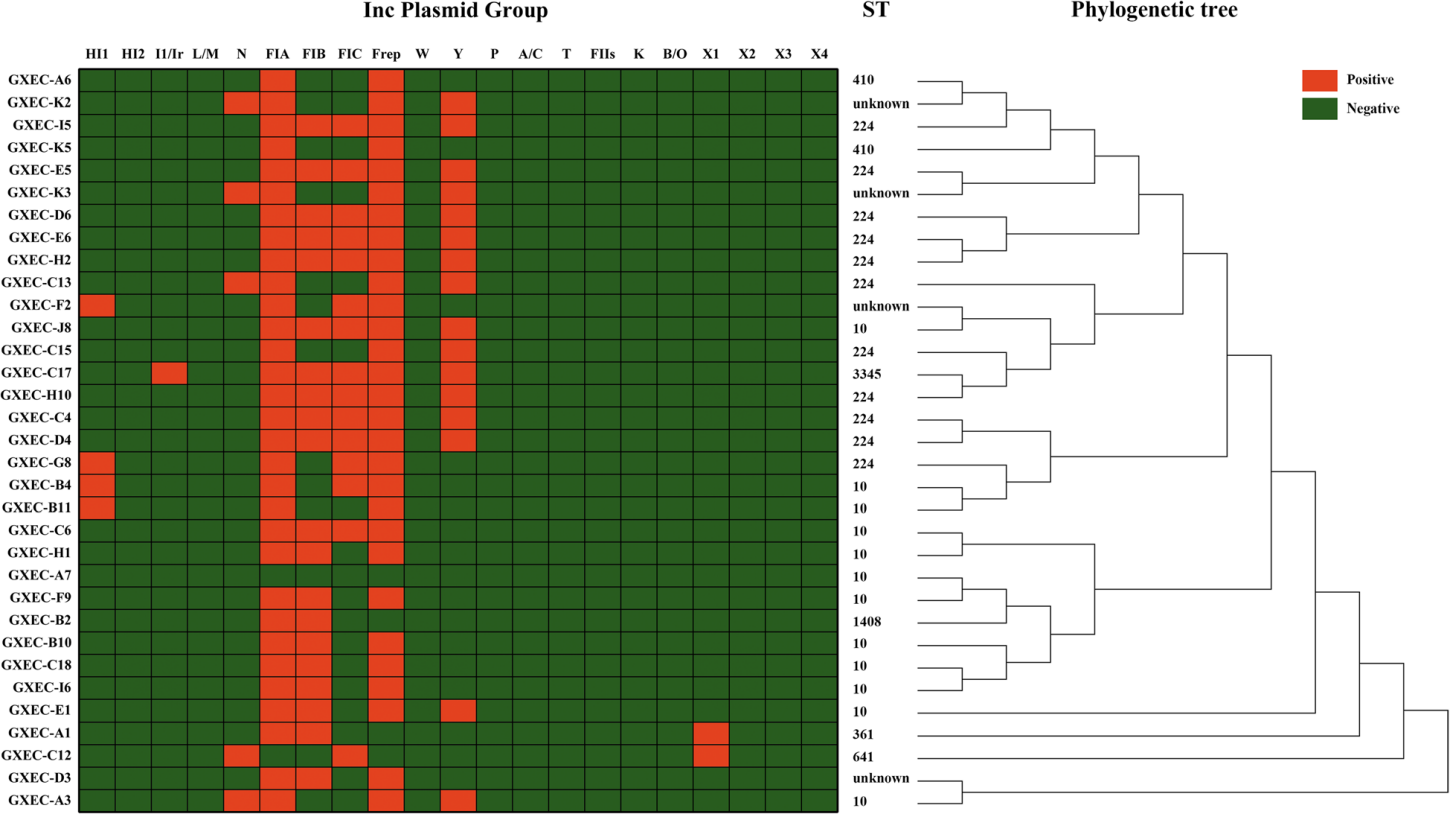
STs, incompatibility plasmid groups and phylogenetic tree of the 33 swine-origin MCRPEC isolates. Phylogenetic tree was generated by Neighbor joining method in MEGA-X, and toggled scaling of tree

The full-length 16S rRNA gene sequences of the 33 MCRPEC strains were used to generate a phylogenetic tree by means of Neighbor Joining method in MEGA-X (Fig. 1). Thirty-three MCRPEC strains were classified into eight distinct STs, including ST10, ST224, ST361, ST410, ST641, ST1408, ST3345, and an unknown ST. ST10 and ST224 were the dominant STs, which accounted for 69.7% (23/33) (Fig. 1). More information about MLST was included in the supplementary materials (supplementary materials Table 2).

Multiple PCR was used to identify incompatibility plasmid groups in MCRPEC by using plasmid DNA of MCRPEC isolates. As is shown in Fig. 1, six incompatibility plasmid groups were detected, including IncFI (97.0%, 32/33), IncHI (12.1%, 4/33), IncY (48.5%, 16/33), IncN (15.2%, 5/33), IncI1 (3.0%, 1/33) and IncX1 (6.1%, 2/33). Ten (30.3%, 10/33) MCRPEC isolates were detected to carry one incompatibility plasmid group (IncFI). Sixteen (16/33, 48.5%) MCRPEC isolates were detected to carry two incompatibility plasmid groups, among which eleven (11/16, 68.8%) isolates were the combination of IncFI and IncY, four (4/16, 25%) isolates displayed the combination of IncHI and IncFI, and one (1/16, 6.2%) isolate exhibited the combination of IncFI and IncX1. Six (6/33, 18.2%) MCRPEC isolates were detected to carry three incompatibility plasmid groups, among which four (4/6, 66.6%) isolates were the combination of IncFI, IncN and IncY, one (1/6, 16.7%) isolate was the combination of IncFI, IncI1 and IncY, and one (1/16, 16.7) isolate was the combination of IncFI, IncN and IncX1. In addition, one (1/33, 3.0%) MCRPEC isolate was not detected to carry incompatibility plasmid group.

### Antimicrobial resistance in MCRPEC

**Fig. 2:**
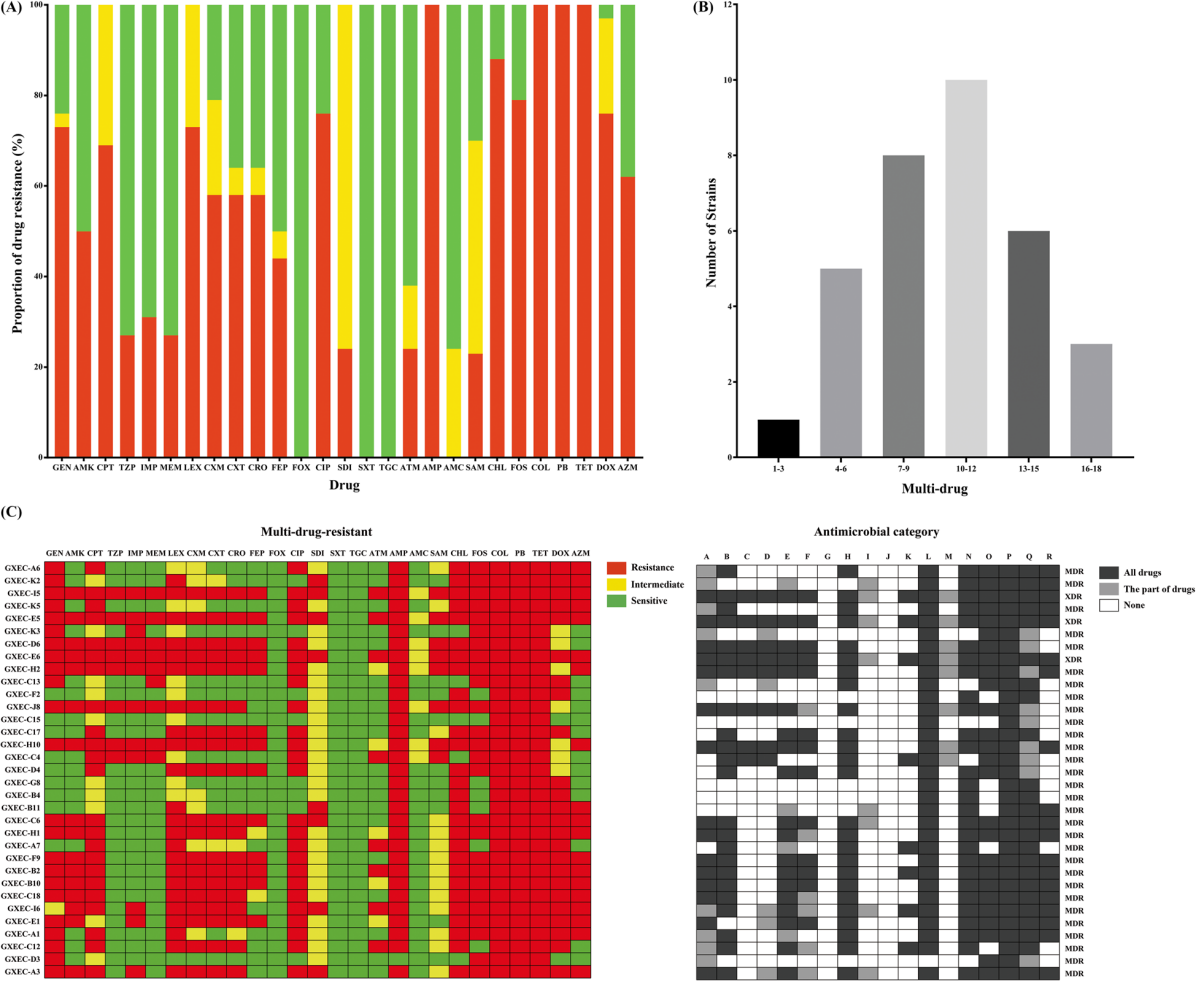
Antimicrobial resistance characteristics of the 33 swine-origin MCRPEC isolates from swine *E. coli*. **a** Antimicrobial resistance proportion, the bar chart showed the percentages of the 33 MCRPEC isolates that were sensitive (green), intermediate (yellow), or resistant (red) to 27 commonly used antimicrobials. **b** Statistics of the multi-antimicrobial category. **c** The first panel consists of 27 columns representing the sensitivity (green), intermediates (yellow), or resistance (red) of 27 antimicrobial agents. The next panel contains 18 columns indicating the antimicrobial category of the 33 MCRPEC isolates, including A: aminoglycosides, B: anti-MRSA cephalosporins, C: antipseudomonal penicillins + β-lactamase inhibitors, D: carbapenems, E: 1st and 2nd generation cephalosporins, F: 3rd and 4th generation cephalosporins, G: cephamycins, H: fluoroquinolones, I: folate pathway inhibitors, J: glycylcyclines, K: monobactams, L: penicillins, M: penicillins + β-lactamase inhibitors, N: phenicols, O: phosphonic acids, P: polymyxins, Q: tetracyclines, and R: Macrolides. The colors indicate resisitance to all kinds agents of one antimicrobial category (black), resistant to some of the agents of one antimicrobial category (gray), no agent (white). The rightmost section are judgments for MDR, XDR, or PDR. Multidrug resistant (MDR) is the acquired (but not natural) insensitivity (mediated or resistant) to three or more antimicrobial agents (at least one in each category). Extensive resistant (XDR) means that it is insensitive to all antimicrobial species (at least one in each category) except for those in the 1–2 category. PDR is defined as non-susceptibility to all agents in all antimicrobial categories. The MICs of antimicrobial resistance test were putted in supplementary material Table 4

The antimicrobial resistance proportion of thirty-three MCRPEC isolates were as follows: gentamicin (72.7%, 24/33), amikacin (48.5%, 16/33), ceftaroline (69.7%, 23/33), piperacillin-tazobactam (24.2%, 8/33), imipenem (36.4%, 12/33), meropenem (24.2%, 8/33), cefalexin (69.7%, 23/33), cefuroxime (57.6%, 19/33), cefotaxime (57.6%, 19/33), ceftriaxone (57.6%, 19/33), cefepime (39.4%, 13/33), cefoxitin (0%, 0/33), ciprofloxacin (75.8%, 25/33), sulfadiazine (24.2%, 8/33), trimethoprim-sulphamethoxazole (0%, 0/33), aztreonam (24.2%, 8/33), ampicillin (97.0%, 32/33), amoxicillin-clavulanic acid (0%, 0/33), ampicillin-sulbactam (24.2%, 8/33), chloramphenicol (84.8%, 28/33), fosfomycin (78.8%, 26/33), tetracycline (100%, 33/33), doxycycline (72.7%, 24/33), azithromycin (57.6%, 19/33), polymyxin B (100%, 33/33) and colistin (100%, 33/33) (Fig. 2a). In addition, each MCRPEC isolate showed significant antimicrobial resistance (Fig. 2b). According to the results of cephalosporin susceptibility test, nineteen strains (57.6%, 19/33) were resistant to the 3rd and 4th generation cephalosporins and twelve strains (36.4%, 12/33) were resistant to carbapenem (Fig. 2a). According to the definition of MDR, XDR, and PDR bacteria, all 33 MCRPEC isolates were identified as MDR (Fig. 2b) [2]. Among them, three of the MDR MCRPEC isolates were identified as XDR (Fig. 2c).

### Coexistence of *mcr-1* gene in plasmids with β-lactamase antimicrobial resistance genes and non-β-lactamase antimicrobial resistance genes in the MCRPEC isolates

**Fig. 3:**
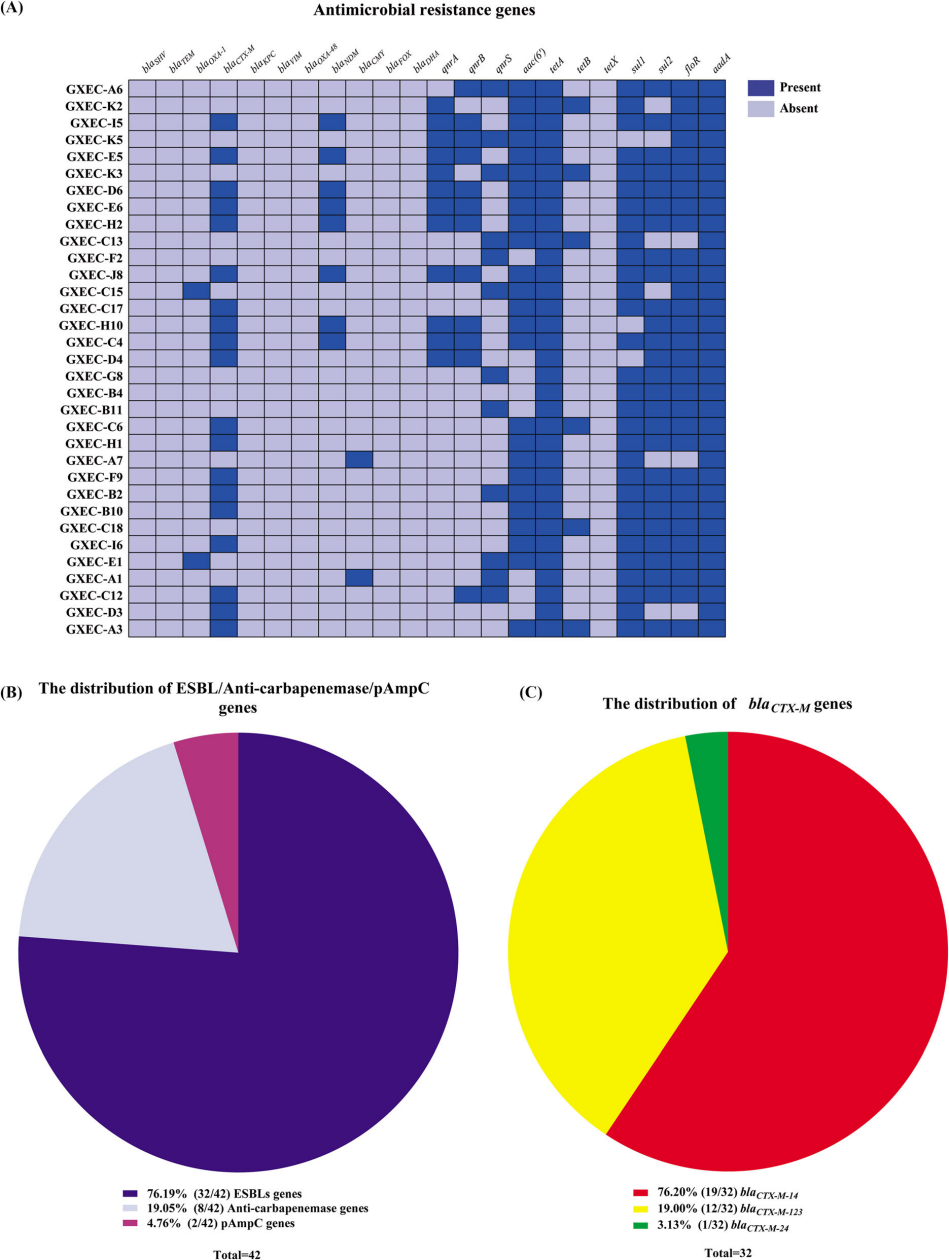
Plasmid-carrying resistant genes of the 33 swine-origin MCRPEC isolates (**a**) The panel includes 22 columns indicating the presence or absence of plasmid-mediated resistance genes. Dark blue means presence, and light Blue means absence. **b** The distribution of the total ESBL/Carbapenemase/pAmpC genes in the MCRPEC isolates. **c** The distribution of *bla*_*CTX-M*_ among thirty-two ESBL genes

There were 22 MCRPEC isolates harbored ESBL genes, including two *bla*_*OXA-1*_ and thirty-two *bla*_*CTX-M*_. The dominate *bla*_*CTX-M*_ gene was *bla*_*CTX-M-14*_ (59.4%, 19/32), followed by *bla*_*CTX-M-123*_ (37.5%, 12/32) and *bla*_*CTX-M-24*_ (3.1%, 1/32) (Fig. 3a, b). Additionally, there were two and eight MCRPEC isolates with *bla*_*CMY-2*_ and *bla*_*NDM-5*_ respectively. (Fig. 3a, c).

Furthermore, many non-β-lactamase antimicrobial resistance genes were also detected in MCPEC isolates, including fluoroquinolone resistance gene, tetracycline resistance genes, sulfanilamide resistance genes, aminoglycoside resistance genes and chloramphenicol resistance genes. As is shown in Fig. 3a, the rates of fluoroquinolone resistance related genes q*nrA*, *qnrB*, *qnrS*, and *aac (6′)-Ib-cr* were 36.4% (12/33), 36.4% (12/33), 33.3% (11/33), and 24.2%(8/33) individually. Tetracycline resistance related genes *tetA*, *tetB*, and *tetX* accounted for 100% (33/33), 18.2% (6/33), and 0% (0/33), respectively. Sulfanilamide resistance determinants *sul1* and *sul2* accounted for 90.0% (30/33) and 78.8% (26/33), respectively. Aminoglycoside resistance related gene *aadA* (100%, 33/33) and chloramphenicol resistance related gene *floR* (100%, 33/33) both have 100% detection rates*.*

Besides, virulence genes (Enterotoxigenic *E. coli* and Shigatoxin-pruducing *E. coli*) of thirty-three MCRPEC isolates from clinical diagnostic were detected. The results were shown in Table 3 of supplementary materials. Twenty-six (26/33, 78.8%) MCRPEC isolates were identified as pathogenic *E. coli*, of which twenty-two (22/26, 84.6%) isolates were identified as Enteroxigenic *E. coli* (ETEC), three (3/26, 11.5%) isolates were identified as Shigatoxin-pruducing *E. coli* (STEC), and one (1/26, 3.9%) isolate was both ETEC and STEC. Fifteen (15/22, 68.2%) ETEC isolates only carried *STb* gene, the rest of ETEC isolates (7/22, 31.8%) carried *STb* and *LT* genes. Three STEC isolates only carried *stx2e* gene. The ETEC/STEC isolate carried *STb*, *LT* and *stx2e* genes.

## Discussion

China became the world’s lar raises and consumes about 500 million pigs a year, nearly half the world’s total, and is the world’s largest consumer of antibiotics [22]. The long term over antibiotics fast-growing of resistance [23]. In the challenge of antibiotic resistance, the Chinese government stipulated the veterinary drug prescription management measures in 2013 [24]. In recent years, plasmid-mediated colistin resistant genes *mcr-1* to *mcr-9* have been found worldwide [7–15]. Although the Chinese government began to tighten regulation of colistin in 2017, MCRPEC remains to be a chronic problem in pig farms [25–27]. Thus, we report extensive antimicrobial resistance and plasmid-carrying resistance genes in MCRPEC from pigs in Guangxi, China.

In this study, MCRPEC was found to be highly resistant to some of the β-lactam and non-β-lactam antibiotics commonly used in human medicine and veterinary medicine, such as penicillin, cephalosporins, fluoroquinolones, aminoglycosides, quinolones, sulfonamides and tetracycline. In addition, rare resistance to carbapenems in animal production such as imipenem and meropenem was also found in MCRPEC. According to veterinary prescription list, imipenem and meropenem are not permitted to use in livestock production. Recently, studies have found that MCRPEC contains a “new Delhi metallo-lactam resistance gene” that is resistant to almost all β-lactam antibiotics but monobactam [28, 29]. Thirty-three MCRPEC isolates were susceptible to tigecycline and cefoxitin. According to veterinary prescription list, tigecycline and cefoxitin are not permitted to use in livestock production [24, 25]. As the same time, Guangxi government published the notice explained tigecycline was special use level and cefoxitin was restricted use level (i.e. ordinary people is difficult to acquire) [30]. The presence of these multidrug-resistant phenotypes suggests that MCRPEC coexists with other drug-resistant genes.

Interestingly, there is evidence that ESBL *E. coli* has a higher level of *mcr-1* than non-ESBL *E. coli*, and the rapid rise in ESBL also significantly increases the selective pressure for colistin resistance [17]. In this study, 63.6% (21/33) of MCRPEC strains were detected to contain different ESBL genes simultaneously. Among them, *bla*_*CTX-M*_ (19/21, 90.5%) was dominant in MCRPEC plasmid mediated drug resistance genes. Among the 32 *bla*_*CTX-M*_ sequences of 19 MCRPEC isolates, *bla*_*CTX-M-14*_ gene had the highest proportion *bla*_*CTX-M*_ gene (59.4%, 19/32), followed by *bla*_*CTX-M-123*_ (37.5%, 12/32) and *bla*_*CTX-M-24*_ (3.1%, 1/32). Notably, all MCRPEC isolates carrying the *bla*_*CTX-M-14*_ genes were resistant to ciprofloxacin (Fig. 3a and c), consistent with another study [31]. Recently, several reports have shown that pig waste not only frequently carries *mcr-1* and *bla*_*NDM*_ but also transfers these genes by affecting the environment around farms and contaminating the food chain [22, 27, 32]. Therefore, the co-existence of *mcr-1* gene with carbapenem-resistant gene *bla*_*NDM-5*_ (8/33, 24.2%) in *E. coli* has drawn our attention to the spread of such superbugs in Guangxi.

*E. coli* is by nature sensitive to almost all clinically relevant antimicrobial agents, but this bacterium has a great capacity to accumulate resistance genes, mainly through horizontal gene transfer [33]. In this study, 11 plasmids carrying non-lactam genes were found in MCRPEC, including fluoroquinolone (*qnrA*, *qnrB*, *qnrS*, *aac (6′)-Ib-cr*), tetracycline (*tetA*, *tetB*), sulfonamide (*sul1*, *sul2*), aminoglycoside (*aadA*), and chloramphenicol (*floR*), suggesting that the effect of MCRPEC on spreading non-β-lactam genes should not be underestimated.

It has previously been reported that *mcr-1* gene was found in the conjugative plasmids, IncI2, IncFII, IncX4, IncHI1, IncHI2, IncP, IncF, and IncY [34, 35]. We also detected these incompatibility types by PCR typing. The 33 MCRPEC isolates showed six different Inc. plasmid groups including IncHI, IncI1, IncN, IncFI, IncY, IncX1. Meanwhile, we found that IncFIA and IncF_repB_ were prevalent in 33 MCRPEC in this study. MLST results reflected that ST10 (13/33, 39.3%) was the most common ST among the 33 MCRPEC isolates, ST224 (10/33, 30.3%) and another ST (10/33, 30.3%) followed (Fig. 1). Recently, a study analyzed 616 whole genomes of *mcr-1-*positive *E. coli* isolates from NCBI online database, among them ST10 was the most abundant MCRPEC strains [36].

## Conclusions

The study showed that thirty-three *mcr-1*-positive *E. coli* isolates in Guangxi had a wide range of antimicrobial resistance. Plasmid-carrying resistance genes might be the main cause of MCRPEC multidrug resistance. The results indicated that many ESBL genes (*bla*_*CTX-M*_, *bla*_*OXA-1*_) coexisted with mcr-1. The carbapenemase gene *bla*_*NDM-5*_ was detected in 8 MCRPEC strains. Furthermore, a number of non-β-lactam genes also coexisted with *mcr-1* gene. Food animals and their feces are important sources of bacterial drug resistance transfer, our study highlights the necessity for long-term surveillance of *mcr-1*-positive *E. coli* in pigs.

## Methods

### Sample collection and detection of MCRPEC isolates

A total of 116 samples were collected from 44 pig farms that include 37 family farms and 7 swine breeding farms distributed in different towns of Guangxi, China in 2018. In which, 51 samples were collected from family farms and 65 samples were collected from swine breeding farms. These samples were collected from June 2018 to December 2018 and taken from rectal swabs or lung, intestinal tract, or lymph gland tissue collected from dead or unhealthy pigs with diarrhea or dyspnea. Before this study, these samples were sent to clinical veterinary laboratory of College of Animal Science and Technology of Guangxi University for molecular diagnosis. These farms managed about 27.8 thousand fattening pigs and 30.8 thousand breeding pigs during this study period. All fattening pigs belonged to family farms which were companies plus farmer model and small in size (number of pigs were between 400 to 1000 in one farm). Swine breeding farms adopted closed management and bigger in size (number of pigs were between 1000 to 5000 in one farm). In supplementary material Table 5, we provided description of 116 samples origin.

First, the collected samples were inoculated with MacConkey agar for 24 h at 37 °C. Then all single colonies of different forms on MacConkey agar were inoculated with eosin-methylene blue agar to screen suspected *E. coli* isolates. Colonies with a purplish black color or a metallic dark green color on the eosin-methylene blue agar were considered as suspected *E. coli* and further inoculated into LB Broth (Luria-Bertani Broth) for 8-10 h at 37 °C. Colistin resistance isolates were isolated by self-made SuperPolymyxin medium (i.e. a mixture of 10 ml Eosin-methylene blue agar and 35 μg colistin) as previous reported [37]. The genome DNA of Colistin resistance isolates was extracted with TIANamp Bacteria DNA Kit. Colistin resistance *E. coli* strains were determined by 16S rRNA gene sequencing and BLAST analysis (i.e. First, DNA and primers were used to amplify target fragment largely. PCR amplicons were purified by agarose gel electrophoresis and gel extraction from TIAN Gel Extraction Kit. Purified PCR products were sequenced by ABI 3730xl DNA Analyzer. SeqMan was used for sequence analysis. Finally, sequences were BLAST in NCBI.) [38]. Phylogenetic tree was generated by Neighbor joining statistical method in MEGA-X software. Thirty-three MCRPEC isolates would be tested in follow-up experiments. These isolates were from 116 pigs (un-weaned piglets, nursery piglets and sows) of 25 pig farms of different sizes distributed in 18 different towns of Guangxi, China in 2018 (Table 1). All *E. coli* isolates were stored in glycerol medium at − 80 °C.

**Table 1:** MCRPEC samples collection information

Number	Town/County/City	Date (Month/Day)	Usage/Scale	Clinical symptom
GXEC-A1	Ning Wu, Wu Ming, Nan Ning	June 11	Porker/800	Diarrhea
GXEC-A3	Gan Xu, Wu Ming, Nan Ning	June 19	Porker/600	Diarrhea
GXEC-A6	Jin Ling, Xi Xiang Tang, Nan Ning	July 05	Porker/570	Diarrhea
GXEC-A7	Shuang Ding, Xi Xiang Tang, Nan Ning	July 14	Porker/400	Diarrhea
GXEC-B2	Shuang Ding, Xi Xiang Tang, Nan Ning	July 23	Porker/700	Diarrhea/Dyspnea
GXEC-B4	Jin Ling, Xi Xiang Tang, Nan Ning	August 02	Porker/800	Diarrhea/Dyspnea
GXEC-B10	Shuang Ding, Xi Xiang Tang, Nan Ning	August 19	Porker/530	Diarrhea
GXEC-B11	Fu cheng, Wu Ming, Nan Ning	August 27	Porker/1019	Diarrhea
GXEC-C4	Shuang Qiao, Wu Ming, Nan Ning	September 28	Porker/750	Diarrhea
GXEC-C6	Ling Tian, Ling Chuan, Gui Lin	October 15	Porker/1000	Diarrhea/Dyspnea
GXEC-C12	Shuang Qiao, Wu Ming, Nan Ning	October 15	Porker/600	Diarrhea
GXEC-C13	Sha Tian, Ping Gui, He Zhou	November 01	Porker/900	Diarrhea/Dyspnea
GXEC-C15	Shuang Ding, Xi Xiang Tang, Nan Ning	October 29	Porker/800	Diarrhea
GXEC-C17	He Jie, Ping Gui, He Zhou	November 17	Porker/900	Diarrhea
GXEC-C18	Ren Yi, Ba Bu, He Zhou	December 01	Porker/900	Diarrhea/Dyspnea
GXEC-D3	Sha Tian, Ping Gui, He Zhou	November 18	Porker/700	Diarrhea
GXEC-D4	Kui Yang, Xing Ye, Yu Lin	December 16	Porker/830	Diarrhea
GXEC-D6	Long Meng, Pu Bei, Qin Zhou	December 20	Porker/750	Diarrhea/Dyspnea
GXEC-E1	Shuang Qiao, Wu Ming, Nan Ning	October 11	Un-weaned piglet/700	Diarrhea
GXEC-E5	Shuang Qiao, Wu Ming, Nan Ning	October 11	Un-weaned piglet/700	Diarrhea
GXEC-E6	Shuang Qiao, Wu Ming, Nan Ning	October 11	Sow/700	Diarrhea
GXEC-F2	Da Hua, He Chi	November 03	Un-weaned piglet/5000	Diarrhea
GXEC-F9	Da Hua, He Chi	November 03	Un-weaned piglet/5000	Diarrhea
GXEC-G8	Fu Cheng, Wu Ming, Nan Ning	November 11	Un-weaned piglet/1500	Diarrhea
GXEC-H1	Lu Zhai, Liu Zhou	November 22	Un-weaned piglet/1500	Diarrhea
GXEC-H2	Lu Zhai, Liu Zhou	November 22	Un-weaned piglet/1500	Diarrhea
GXEC-H10	Lu Zhai, Liu Zhou	November 22	Sow/1500	Diarrhea
GXEC-I5	Feng Huang, Xing Bin, Lai bin	December 06	Sow/5000	Diarrhea
GXEC-I6	Feng Huang, Xing Bin, Lai bin	December 06	Sow/5000	Diarrhea
GXEC-J8	Xing An, Xing An, Gui Lin	December 15	Un-weaned piglet/1500	Diarrhea
GXEC-K2	San Jie, Ling Chuan, Gui Lin	December 22	Un-weaned piglet/1000	Diarrhea
GXEC-K3	San Jie, Ling Chuan, Gui Lin	December 22	Un-weaned piglet/1000	Diarrhea
GXEC-K5	San Jie, Ling Chuan, Gui Lin	December 22	Sow/1000	Diarrhea

Un-weaned piglets were concentrated on less than 20 days. Nursery piglets were concentrated on 40 to 60 days. Sows were less than 100 days old

The MCRPEC isolates were preliminarily screened by PCR amplification using genome DNA and special primer pairs for the *mcr-1*, *mcr-2*, *mcr-3*, *mcr-4*, *mcr-5*, *mcr-6*, *mcr-7*, and *mcr-8* genes (supplementary material Tables 1), [39, 40]. To identify MCRPEC strains, the *mcr-1* gene sequences in the *E. coli* strains were determined by direct sequencing from the PCR products and BLAST analysis [38].

### Detection of multilocus sequence typing (MLST) and incompatibility plasmid groups

MLST analysis was performed by PCR amplicons of seven housekeeping genes, namely *adk*, *fumC*, *gyrB*, *icd*, *mdh*, *purA*, and *recA* using genome DNA. PCR amplicons were sequenced after purified by agarose gel electrophoresis and gel extraction by TIAN Gel Extraction Kit. The gene sequences for seven housekeeping genes were uploaded to the EnteroBase database to obtain the sequence type (ST) of corresponding *E. coli* isolate [41].

Incompatibility plasmid groups were assigned by PCR-based replicon types (HI1, HI2, I1, L/M, N, FIA, FIB, FIC, Frep, W Y, P, A/C, T, FIIS, K/B, B/O) [42]. Additional PCRs were performed for the IncX (X1, X2, X3, X4) replicon types [43]. The primers of house genes and replicon, and PCR reaction conditions were included in supplementary material Table 1.

### Antimicrobial susceptibility testing

According to the European Society of Clinical Microbiology and Infectious Diseases (ESCMID) consensus, a total of 27 commonly used human antimicrobials from 18 antimicrobial categories were selected in this study, including gentamicin, amikacin, ceftaroline, piperacillin-tazobactam, imipenem, meropenem, cefalexin, cefuroxime, cefotaxime, ceftriaxone, cefepime, cefoxitin, ciprofloxacin, sulfadiazine, trimethoprim-sulphamethoxazole, aztreonam, ampicillin, amoxicillin-clavulanic acid, ampicillin-sulbactam, chloramphenicol, fosfomycin, tetracycline, doxycycline, azithromycin, polymyxin B and colistin (Table 2), [2]. Minimum inhibitory concentrations (MICs) were determined by using the agar microdilution (Mueller-Hinton Agar) method according to the Clinical and Laboratory Standards Institute [44]. The MICs of each drug were measured and recorded. *E. coli* ATCC25922 was used as a quality control. Resistant breakpoints of other antimicrobial abided by the CLSI-M100 document [45]. CLSI breakpoints are not available for colistin and cefalexin. So, in this study, we adopted the European Committee on Antimicrobial Susceptibility Testing Resistant/Susceptible breakpoints for determine colistin and cefalexin MICs. MICs of ≤2 mg/L and ≤ 16 mg/L are considered as susceptible (S) for colistin and cephalexin, respectively, according to the EUCAST guidelines [46].

**Table 2:** The antimicrobial agents for 17 antimicrobial categories used to define the *E. coli* antimicrobial resistance

Antimicrobial category	Antimicrobial agent
Aminoglycosides	Gentamicin (GEN) Amikacin (AMK)
Anti-MRSA cephalosporin	Ceftaroline (CPT)
Antipseudomonal penicillin + β-lactamase inhibitor	Piperacillin/tazobactam (TZP)
Carbapenem	Imipenem (IMP) Meropenem (MEM)
Non-extended spectrum cephalosporins	Cefalexin (LEX) Cefuroxime (CXM)
3rd and 4th generation cephalosporins	Cefotaxime (CTX) Ceftriaxone (CRO) Cefepime (FEP)
Cephamycin	Cefoxitin (FOX)
Fluoroquinolone	Ciprofloxacin (CIP)
Folate pathway inhibitor	Sulfadiazine (SDI) Trimethoprim/sulfamethoxazole (SXT)
Glycylcycline	Tigecycline (TGC)
Monobactam	Aztreonam (ATM)
Penicillin	Ampicillin (AMP)
Penicillin+β-lactamase inhibitors	Amoxicillin/clavulanicacid (AMC) Ampicillin-sulbactam (SAM)
Phenicol	Chloramphenicol (CHL)
Phosphonic acid	Fosfomycin (FOS)
Polymyxins	PolymyxinB (PB) Colistin (COL)
Tetracycline	Tetracycline (TET) Doxycycline (DOX)
Macrolides	Azithromycin (AZM)

### Molecular identification of ESBL, pAmpC, and carbapenem resistance genes

The ESBL, plasmid-mediated AmpC (pAmpC), and carbapenem genes were detected by multiplex PCR in plasmid DNA of MCRPEC isolates. The ESBL genes (*bla*_*CTX-M*_, *bla*_*TEM*_, *bla*_*OXA-1*_, and *bla*_*SHV*_), plasmid-mediated AmpC (pAmpC) genes (*bla*_*CMY*_, *bla*_*FOX*_, *bla*_*DHA*_), and carbapenem resistance genes (*bla*_*NDM*_, *bla*_*KPC*_, *bla*_*OXA-48*_, and *bla*_*IMP*_) were amplified using specific primers, as previously reported [47]. The DNA sequences for the ESBL, plasmid-mediated AmpC (pAmpC), and carbapenemase genes were determined by using BLAST analysis [38]. The primers of β-lactamase resistance genes and PCR reaction conditions were included in supplementary material Table 1.

### Detection of non-β-lactamase antimicrobial resistance genes

The non-β-lactamase antimicrobial resistance genes were detected by PCR in plasmid DNA of MCRPEC isolates. The special primers included plasmid-encoded fluoroquinolone resistance genes (*qnrA*, *qnrB*, *qnrS*, *aac (6′)-Ib-cr*) [48], tetracycline resistance genes (*tetA*, *tetB*, *tetX*) [49], sulfonamide resistance genes (*sul1*, *sul2*), aminoglycoside resistance genes (*aadA*), and chloramphenicol resistance genes (*floR*), respectively. The primers of non-β-lactamase resistance genes and PCR reaction conditions were included in supplementary material Table 1.

## Supplementary Information

**Additional file 1.**

## Data Availability

The datasets generated and/or analysed during the current study are available in the [Github] repository, [https://github.com/YuanJZ1994/raw-data.git]. All data generated or analysed during this study are included in this published article [and its supplementary information files].

## Abbreviations

MCRPEC: *Mcr-1-positive Escherichia coli*
MDR: Multi-drug-resistance
XDR: Extensively-drug-resistant
PDR: Pan-drug-resistant
CREC: Carbapenem resistant *Escherichia coli*
ESBL: Extended-spectrum β-lactam
pAmpC: Plasmid-mediated AmpC
