# Supplementary material for: Extensive antimicrobial resistance and plasmid-carrying resistance genes in mcr-1-positive E. coli sampled in swine, in Guangxi, South China
Source: BMC Vet Res. 2021 Feb 18;17:86. doi: 10.1186/s12917-021-02758-4 (PMC7893886; doi:10.1186/s12917-021-02758-4)
Supplement: Supplementary file 1 — Additional file 1. [file 12917_2021_2758_MOESM1_ESM.docx]

**Supplementary material**

| **Table 1** Primers in this study | | | | | |
| --- | --- | --- | --- | --- | --- |
| Primer categories | Primer name | Sequence (5’ to 3’) | Amplicon size (bp) | annealing temperature (Tm) | Reference |
| House genes | adk-F | ATTCTGCTTGGCGCTCCGGG | 583 | 54 | <https://pubmlst.org/bigsdb?db=pubmlst_escherichia_seqdef&page=downloadAlleles&tree=1> |
|  | adk-R | CCGTCAACTTTCGCGTATTT |  |  |  |
|  | fumC-F | TCACAGGTCGCCAGCGCTTC | 806 | 54 |  |
|  | fumC-R | GTACGCAGCGAAAAAGATTC |  |  |  |
|  | gyrB-F | TCGGCGACACGGATGACGGC | 911 | 60 |  |
|  | gyrB-R | ATCAGGCCTTCACGCGCATC |  |  |  |
|  | icd-F | ATGGAAAGTAAAGTAGTTGTTCCGGCACA | 878 | 54 |  |
|  | icd-R | GGACGCAGCAGGATCTGTT |  |  |  |
|  | mdh-F | ATGAAAGTCGCAGTCCTCGGCGCTGCTGGCGG | 932 | 60 |  |
|  | mdh-R | TTAACGAACTCCTGCCCCAGAGCGATATCTTTCTT |  |  |  |
|  | purA-F | CGCGCTGATGAAAGAGATGA | 816 | 54 |  |
|  | purA-R | CATACGGTAAGCCACGCAGA |  |  |  |
|  | recA-F | CGCATTCGCTTTACCCTGACC | 780 | 58 |  |
|  | recA-R | TCGTCGAAATCTACGGACCGGA |  |  |  |
| Plasmid replicon | HI1-F | GGAGCGATGGATTACTTCAGTAC | 471 | 60 | [1] |
|  | HI1-R | TGCCGTTTCACCTCGTGAGTA |  |  |  |
|  | HI2-F | TTTCTCCTGAGTCACCTGTTAACAC | 644 | 60 |  |
|  | HI2-R | GGCTCACTACCGTTGTCATCCT |  |  |  |
|  | I1-F | CGAAAGCCGGACGGCAGAA | 139 | 60 |  |
|  | I1-R | TCGTCGTTCCGCCAAGTTCGT |  |  |  |
|  | L/M-F | GGATGAAAACTATCAGCATCTGAAG | 785 | 60 |  |
|  | L/M-R | CTGCAGGGGCGATTCTTTAGG |  |  |  |
|  | N-F | GTCTAACGAGCTTACCGAAG | 559 | 60 |  |
|  | N-R | GTTTCAACTCTGCCAAGTTC |  |  |  |
|  | FIA-F | CCATGCTGGTTCTAGAGAAGGTG | 462 | 60 |  |
|  | FIA-R | GTATATCCTTACTGGCTTCCGCAG |  |  |  |
|  | FIB-F | GGAGTTCTGACACACGATTTTCTG | 702 | 60 |  |
|  | FIB-R | CTCCCGTCGCTTCAGGGCATT |  |  |  |
|  | W-F | CCTAAGAACAACAAAGCCCCCG | 242 | 60 |  |
|  | W-R | GGTGCGCGGCATAGAACCGT |  |  |  |
|  | Y-F | AATTCAAACAACACTGTGCAGCCTG | 765 | 60 |  |
|  | Y-R | GCGAGAATGGACGATTACAAAACTTT |  |  |  |
|  | P-F | CTATGGCCCTGCAAACGCGCCAGAAA | 534 | 60 |  |
|  | P-R | TCACGCGCCAGGGCGCAGCC |  |  |  |
|  | FIC-F | GTGAACTGGCAGATGAGGAAGG | 262 | 60 |  |
|  | FIC-R | TTCTCCTCGTCGCCAAACTAGAT |  |  |  |
|  | A/C-F | GAGAACCAAAGACAAAGACCTGGA | 465 | 60 |  |
|  | A/C-R | ACGACAAACCTGAATTGCCTCCTT |  |  |  |
|  | T-F | TTGGCCTGTTTGTGCCTAAACCAT | 750 | 60 |  |
|  | T-R | CGTTGATTACACTTAGCTTTGGAC |  |  |  |
|  | FII_s_-F | CTGTCGTAAGCTGATGGC | 270 | 60 |  |
|  | FII_s_-R | CTCTGCCACAAACTTCAGC |  |  |  |
|  | F_repB_-F | TGATCGTTTAAGGAATTTTG | 270 | 52 |  |
|  | F_repB_-R | GAAGATCAGTCACACCATCC |  |  |  |
|  | K/B-F | GCGGTCCGGAAAGCCAGAAAAC | 160 | 60 |  |
|  | K-R | TCTTTCACGAGCCCGCCAAA |  |  |  |
|  | B/O-R | TCTGCGTTCCGCCAAGTTCGA | 159 | 60 |  |
|  | X1-F | GCTTAGACTTTGTTTTATCGTT | 461 | 62 | [2] |
|  | X1-R | TAATGATCCTCAGCATGTGAT |  |  |  |
|  | X2-F | GCGAAGAAATCAAAGAAGCTA | 678 | 63 |  |
|  | X2-R | TGTTGAATGCCGTTCTTGTCCAG |  |  |  |
|  | X3-F | GTTTTCTCCACGCCCTTGTTCA | 351 | 63 |  |
|  | X3-R | CTTTGTGCTTGGCTATCATAA |  |  |  |
|  | X4-F | AGCAAACAGGGAAAGGAGAAGACT | 569 | 62 |  |
|  | X4-R | TACCCCAAATCGTAACCTG |  |  |  |
| Carbapenem resistance genes | *bla_NDM_*-F | GGTTTGGCGATCTGGTTTTC | 621 | 55 | [3] |
|  | *bla_NDM_*-R | CGGAATGGCTCATCACGATC |  |  |  |
|  | *bla_KPC_*-F | CGTCTAGTTCTGCTGTCTTG | 566 | 55 |  |
|  | *bla_KPC_*-R | CTTGTCATCCTTGTTAGGCG |  |  |  |
|  | *bla_OXA-48_*-F | GCGTGGTTAAGGATGAACAC | 438 | 55 |  |
|  | *bla_OXA-48_*-R | CATCAAGTTCAACCCAACCG |  |  |  |
|  | *bla_IMP_*-F | GGAATAGAGTGGCTTAAYTCTC | 232 | 55 |  |
|  | *bla_IMP_*-R | GGTTTAAYAAAACAACCACC |  |  |  |
| ESBL genes | *bla_SHV_*-F | AGCCGCTTGAGCAAATTAAAC | 713 | 60 | [4] |
|  | *bla_SHV_*-R | ATCCCGCAGATAAATCACCAC |  |  |  |
|  | bla_TEM_-F | CATTTCCGTGTCGCCCTTATTC | 800 | 60 |  |
|  | bla_TEM_-R | CGTTCATCCATAGTTGCCTGAC |  |  |  |
|  | *bla_OXA-1_*-F | GGCACCAGATTCAACTTTCAAG | 564 | 60 |  |
|  | *bla_OXA-1_*-R | GACCCCAAGTTTCCTGTAAGTG |  |  |  |
|  | *bla_CTX-M_* group 1-F | TTAGGAARTGTGCCGCTGYA | 688 | 60 |  |
|  | *bla_CTX-M_* group 1-R | CGATATCGTTGGTGGTRCCAT |  |  |  |
|  | *bla_CTX-M_* group 2-F | CGTTAACGGCACGATGAC | 404 | 60 |  |
|  | *bla_CTX-M_* group 2-R | CGATATCGTTGGTGGTRCCAT-3’ |  |  |  |
|  | *bla_CTX-M_* group 9-F | TCAAGCCTGCCGATCTGGT | 561 | 60 |  |
|  | *bla_CTX-M_* group 9-R | TGATTCTCGCCGCTGAAG |  |  |  |
| Plasmid-mediated AmpC genes | *bla_CMY_*-F | CGAAGAGGCAATGACCAGAC | 895 | 60 |  |
|  | *bla_CMY_*-R | ACGGACAGGGTTAGGATAGY |  |  |  |
|  | *bla_FOX_*-F | CTACAGTGCGGGTGGTTT | 162 | 60 |  |
|  | *bla_FOX_*-R | CTATTTGCGGCCAGGTGA |  |  |  |
|  | *bla_DHA_*-F | TGATGGCACAGCAGGATATTC | 997 | 60 |  |
|  | *bla_DHA_*-R | GCTTTGACTCTTTCGGTATTCG |  |  |  |
| Tetracyclines resistance genes | tet(A)-F* | GCTACATCCTGCTTGCCTTC | 210 | 59.5 | This study |
|  | tet(A)-R* | CATAGATCGCCGTGAAGAGG |  |  |  |
|  | tet(B)-F* | TTGGTTAGGGGCAAGTTTTG | 659 | 59.5 |  |
|  | tet(B)-R* | GTAATGGGCCAATAACACCG |  |  |  |
|  | tet(X)-F | GGAAACCGGCTAATGGCAT | 230 | 55 | [5] |
|  | tet(X)-R | AATCCTACAAATGACAACGTCG |  |  |  |
| Sulfonamide resistance genes | sul1-F* | TCGGACAGGGCGTCTAAG | 925 | 63 | This study |
|  | sul1-R* | GGGTATCGGAGCGTTTGC |  |  |  |
|  | sul2-F* | CTTGTTTCGTCCGACACAGA | 435 | 60 |  |
|  | sul2-R* | GAAGCGCAGCCGCAATTCAT |  |  |  |
| Aminoglycosides resistance genes | aadA1-F* | GCAGCGCAATGACATTCTTG | 282 | 60 | This study |
|  | aadA1-R* | ATCCTCGGCGCGATTTTG |  |  |  |
| Quinolones resistance genes | qnrA-F | AGAGGATTTCTCACGCCAGG | 580 | 54 | [6] |
|  | qnrA-R | TGCCAGGCACAGATCTTGAC |  |  |  |
|  | qnrB-F | GGMATHGAAATTCGCCACTG | 264 | 54 |  |
|  | qnrB-R | TTTGCYGYYCGCCAGTCGAA |  |  |  |
|  | qnrS-F | GCAAGTTCATTGAACAGGGT | 428 | 54 |  |
|  | qnrS-R | TCTAAACCGTCGAGTTCGGCG |  |  |  |
|  | *aac(6’)-Ib-cr*-F | TTGCGATGCTCTATGAGTGGCTA | 482 | 55 | [7] |
|  | *aac(6’)-Ib-cr*-R | CTCGAATGCCTGGCGTGTTT |  |  |  |
| Chloramphenicol resistance genes | folR-F* | GAACACGACGCCCGCTAT | 601 | 54 | This study |
|  | folR-R* | TTCCGCTTGGCCTATGAG |  |  |  |
| Colistin resistance genes | mcr-1-F | AGTCCGTTTGTTCTTGTGGC | 320 | 55 | [8] |
|  | mcr-1-R | AGATCCTTGGTCTCGGCTTG |  |  |  |
|  | mcr-2-F | CAAGTGTGTTGGTCGCAGTT | 715 | 55 |  |
|  | mcr-2-R | TCTAGCCCGACAAGCATACC |  |  |  |
|  | mcr-3-F | AAATAAAAATTGTTCCGCTTATG | 929 | 55 |  |
|  | mcr-3-R | AATGGAGATCCCCGTTTTT |  |  |  |
|  | mcr-4-F | TCACTTTCATCACTGCGTTG | 1116 | 55 |  |
|  | mcr-4-R | TTGGTCCATGACTACCAATG |  |  |  |
|  | mcr-5-F | ATGCGGTTGTCTGCATTTATC | 1644 | 55 |  |
|  | mcr-5-R | TCATTGTGGTTGTCCTTTTCTG |  |  |  |
|  | mcr-6-F | GTCCGGTCAATCCCTATCTGT | 556 | 55 | [9] |
|  | mcr-6-R | ATCACGGGATTGACATAGCTAC |  |  |  |
|  | mcr-7-F | TGCTCAAGCCCTTCTTTTCGT | 892 | 55 |  |
|  | mcr-7-R | TTCATCTGCGCCACCTCGT |  |  |  |
|  | mcr-8-F | AACCGCCAGAGCACAGAATT | 667 | 60 |  |
|  | mcr-8-R | TTCCCCCAGCGATTCTCCAT |  |  |  |

Negative control: total plasmid DNA of ATCC25922

Positive control: * means using resistance gene-positive plasmid as positive control, other PCR products was sequenced through sanger.

**References**

1. Carattoli A, Bertini A, Villa L, Falbo V, Hopkins KL, Threlfall EJ. **Identification of plasmids by PCR-based replicon typing**. *J Microbiol Methods* 2005, **63**(3):219-228.

2. Johnson TJ, Bielak EM, Fortini D, Hansen LH, Hasman H, Debroy C, Nolan LK, Carattoli A. **Expansion of the IncX plasmid family for improved identification and typing of novel plasmids in drug-resistant Enterobacteriaceae**. *Plasmid* 2012, **68**(1):43-50.

3. Poirel L, Walsh TR, Cuvillier V, Nordmann P. **Multiplex PCR for detection of acquired carbapenemase genes**. *Diagn Microbiol Infect Dis* 2011, **70**(1):119-123.

4. Dallenne C, Da Costa A, Decre D, Favier C, Arlet G. **Development of a set of multiplex PCR assays for the detection of genes encoding important beta-lactamases in Enterobacteriaceae**. *J Antimicrob Chemother* 2010, **65**(3):490-495.

5. He T, Wang R, Liu D, Walsh TR, Zhang R, Lv Y, Ke Y, Ji Q, Wei R, Liu Z *et al*. **Emergence of plasmid-mediated high-level tigecycline resistance genes in animals and humans**. *Nat Microbiol* 2019, **4**(9):1450-1456.

6. Cattoir V, Poirel L, Rotimi V, Soussy CJ, Nordmann P. **Multiplex PCR for detection of plasmid-mediated quinolone resistance qnr genes in ESBL-producing enterobacterial isolates**. *J Antimicrob Chemother* 2007, **60**(2):394-397.

7. Park CH, Robicsek A, Jacoby GA, Sahm D, Hooper DC. **Prevalence in the United States of aac(6')-Ib-cr encoding a ciprofloxacin-modifying enzyme**. *Antimicrob Agents Chemother* 2006, **50**(11):3953-3955.

8. Rebelo AR, Bortolaia V, Kjeldgaard JS, Pedersen SK, Leekitcharoenphon P, Hansen IM, Guerra B, Malorny B, Borowiak M, Hammerl JA *et al*. **Multiplex PCR for detection of plasmid-mediated colistin resistance determinants, mcr-1, mcr-2, mcr-3, mcr-4 and mcr-5 for surveillance purposes**. *Euro Surveill* 2018, **23**(6).

9. Yang F, Shen C, Zheng X, Liu Y, El-Sayed Ahmed MAE, Zhao Z, Liao K, Shi Y, Guo X, Zhong R *et al*. **Plasmid-mediated colistin resistance gene mcr-1 in Escherichia coli and Klebsiella pneumoniae isolated from market retail fruits in Guangzhou, China**. *Infect Drug Resist* 2019, **12**:385-389.

| **Table 2** Information of MLST | | | | | | | | |
| --- | --- | --- | --- | --- | --- | --- | --- | --- |
| Strains | ST | House Genes | | | | | | |
|  |  | adk | fumC | gyrB | icd | mdh | purA | recA |
| GXEC-A1 | 361 | 10 | 99 | 5 | 91 | 8 | 7 | 2 |
| GXEC-A3 | 10 | 10 | 11 | 4 | 8 | 8 | 8 | 2 |
| GXEC-A6 | 410 | 6 | 4 | 12 | 1 | 20 | 18 | 7 |
| GXEC-A7 | 1408 | 4 | 7 | 5 | 220 | 8 | 8 | 2 |
| GXEC-B2 | 10 | 10 | 11 | 4 | 8 | 8 | 8 | 2 |
| GXEC-B4 | 10 | 10 | 11 | 4 | 8 | 8 | 8 | 2 |
| GXEC-B10 | 10 | 10 | 11 | 4 | 8 | 8 | 8 | 2 |
| GXEC-B11 | 10 | 10 | 11 | 4 | 8 | 8 | 8 | 2 |
| GXEC-C4 | 224 | 6 | 4 | 33 | 16 | 11 | 8 | 6 |
| GXEC-C6 | 10 | 10 | 11 | 4 | 8 | 8 | 8 | 2 |
| GXEC-C12 | 641 | 9 | 6 | 33 | 131 | 24 | 8 | 7 |
| GXEC-C13 | unknown | 6 | 252 | 4 | 10 | 7 | 8 | 6 |
| GXEC-C15 | 3345 | 64 | 7 | 1 | 369 | 8 | 8 | 6 |
| GXEC-C17 | 224 | 6 | 4 | 33 | 16 | 8 | 8 | 6 |
| GXEC-C18 | 10 | 10 | 11 | 4 | 8 | 8 | 8 | 2 |
| GXEC-D3 | unknown | 6 | 252 | 4 | 10 | 7 | 8 | 6 |
| GXEC-D4 | 224 | 6 | 4 | 33 | 16 | 11 | 8 | 6 |
| GXEC-D6 | 224 | 6 | 4 | 33 | 16 | 11 | 8 | 6 |
| GXEC-E1 | 10 | 10 | 11 | 4 | 10 | 8 | 8 | 2 |
| GXEC-E5 | 224 | 6 | 4 | 33 | 16 | 11 | 8 | 6 |
| GXEC-E6 | 224 | 6 | 4 | 33 | 16 | 11 | 8 | 6 |
| GXEC-F2 | 10 | 10 | 11 | 4 | 8 | 8 | 8 | 2 |
| GXEC-F9 | 10 | 10 | 11 | 4 | 8 | 8 | 8 | 2 |
| GXEC-G8 | 10 | 10 | 11 | 4 | 8 | 8 | 8 | 2 |
| GXEC-H1 | 10 | 10 | 11 | 4 | 8 | 8 | 8 | 2 |
| GXEC-H2 | 224 | 6 | 4 | 33 | 16 | 11 | 8 | 6 |
| GXEC-H10 | 224 | 6 | 4 | 33 | 16 | 11 | 8 | 6 |
| GXEC-I5 | 224 | 6 | 4 | 33 | 16 | 11 | 8 | 6 |
| GXEC-I6 | 10 | 10 | 11 | 4 | 8 | 8 | 8 | 2 |
| GXEC-J8 | 224 | 6 | 4 | 33 | 16 | 11 | 8 | 6 |
| GXEC-K2 | unknown | 6 | 252 | 4 | 10 | 7 | 8 | 6 |
| GXEC-K3 | unknown | 8 | 252 | 4 | 10 | 7 | 8 | 6 |
| GXEC-K5 | 410 | 6 | 4 | 12 | 1 | 20 | 18 | 7 |

| **Table 3** ETEC/STEC virulence genes test of MCRPEC | | | | | | | |
| --- | --- | --- | --- | --- | --- | --- | --- |
| Strains | ETEC | | | STEC | | | Identity |
|  | STa | STb | LT | stx1 | stx2 | stx2e |  |
| GXEC-A6 |  | **+** |  |  |  |  | ETEC |
| GXEC-K2 |  |  |  |  |  | **+** | STEC |
| GXEC-I5 |  |  |  |  |  |  |  |
| GXEC-K5 |  |  |  |  |  |  |  |
| GXEC-E5 |  | **+** |  |  |  |  | ETEC |
| GXEC-K3 |  | **+** |  |  |  |  | ETEC |
| GXEC-D6 |  | **+** | **+** |  |  | **+** | ETEC/STEC |
| GXEC-E6 |  | **+** |  |  |  |  | ETEC |
| GXEC-H2 |  | **+** | **+** |  |  |  | ETEC |
| GXEC-C13 |  | **+** |  |  |  |  | ETEC |
| GXEC-F2 |  | **+** |  |  |  |  | ETEC |
| GXEC-J8 |  | **+** | **+** |  |  |  | ETEC |
| GXEC-C15 |  | **+** |  |  |  |  | ETEC |
| GXEC-C17 |  | **+** | **+** |  |  |  | ETEC |
| GXEC-H10 |  |  |  |  |  |  |  |
| GXEC-C4 |  | **+** |  |  |  |  | ETEC |
| GXEC-D4 |  | **+** | **+** |  |  |  | ETEC |
| GXEC-G8 |  |  |  |  |  |  |  |
| GXEC-B4 |  | **+** |  |  |  |  | ETEC |
| GXEC-B11 |  | **+** | **+** |  |  |  | ETEC |
| GXEC-C6 |  | **+** |  |  |  |  | ETEC |
| GXEC-H1 |  | **+** |  |  |  |  | ETEC |
| GXEC-A7 |  |  |  |  |  | **+** | STEC |
| GXEC-F9 |  | **+** |  |  |  |  | ETEC |
| GXEC-B2 |  |  |  |  |  | **+** | STEC |
| GXEC-B10 |  |  |  |  |  |  |  |
| GXEC-C18 |  | **+** | **+** |  |  |  | ETEC |
| GXEC-I6 |  |  |  |  |  |  |  |
| GXEC-E1 |  | **+** |  |  |  |  | ETEC |
| GXEC-A1 |  |  |  |  |  |  |  |
| GXEC-C12 |  | **+** |  |  |  |  | ETEC |
| GXEC-D3 |  | **+** | **+** |  |  |  | ETEC |
| GXEC-A3 |  | **+** |  |  |  |  | ETEC |
| Statistics | 69.7% (23/33) | | | 9.1% (4/33) | | | 78.8% (26/33) |

Note: ETEC was Enterotoxigenic *E. coli*. STEC was Shigatoxin-pruducing *E. coli.*

**Table 4** MICs of antimicrobial resistance test (one)

| Strains | MICs（mg/L） | | | | | | | | | | | | | | | | | | | | | |
| --- | --- | --- | --- | --- | --- | --- | --- | --- | --- | --- | --- | --- | --- | --- | --- | --- | --- | --- | --- | --- | --- | --- |
|  | COL | | PB | | IMP | | MEM | | LEX | | CXM | | CTX | | CRO | | FEP | | CPT | | ATM | |
| GXEC-A1 | 4 | R | 2 | R | 2 | S |  | S | 32 | R | 8 | I | 0.5 | S | 2 | I | 2 | S | 8 | R | 4 | S |
| GXEC-A3 | 8 | R | 4 | R | 8 | R |  | S | 32 | R | 64 | R | 8 | R | 8 | R | 2 | S | 8 | R | 4 | S |
| GXEC-A6 | 8 | R | 4 | R | 1 | S |  | S | 16 | R | 16 | I |  | S |  | S |  | S | 8 | R | 4 | S |
| GXEC-A7 | 4 | R | 2 | R | 2 | S |  | S | 32 | R | 8 | I | 2 | I | 2 | I |  | S | 8 | R | 16 | R |
| GXEC-B2 | 8 | R | 4 | R | 2 | S |  | S | 32 | R | 64 | R | 8 | R | 8 | R | 32 | R | 8 | R | 16 | R |
| GXEC-B4 | 8 | R | 4 | R | 2 | S |  | S | 8 | I | 16 | I |  | S |  | S |  | S | 1 | I |  | S |
| GXEC-B10 | 8 | R | 2 | R | 2 | S |  | S | 32 | R | 64 | R | 8 | R | 8 | R | 32 | R | 8 | R | 8 | I |
| GXEC-B11 | 8 | R | 8 | R | 2 | S |  | S | 32 | R | 8 | I | 0.5 | S |  | S |  | S | 1 | I |  | S |
| GXEC-C4 | 4 | R | 2 | R | 16 | R |  | S | 8 | I | 4 | S |  | S | 0.5 | S |  | S | 8 | R | 32 | R |
| GXEC-C6 | 8 | R | 4 | R | 2 | S |  | S | 32 | R | 64 | R | 8 | R | 8 | R | 32 | R | 8 | R | 4 | S |
| GXEC-C12 | 4 | R | 2 | R | 1 | S |  | S | 32 | R | 64 | R | 8 | R | 8 | R |  | S | 8 | R | 32 | R |
| GXEC-C13 | 8 | R | 2 | R | 2 | S |  | S | 8 | I | 4 | S |  | S |  | S |  | S | 1 | I |  | S |
| GXEC-C15 | 8 | R | 2 | R | 1 | S |  | S | 8 | I | 4 | S |  | S |  | S |  | S | 1 | I |  | S |
| GXEC-C17 | 8 | R | 4 | R | 2 | S |  | S | 32 | R | 64 | R | 8 | R | 8 | R | 32 | R | 8 | R | 4 | S |
| GXEC-C18 | 8 | R | 2 | R | 2 | S |  | S | 32 | R | 64 | R | 8 | R | 8 | R | 8 | I | 8 | R |  | S |
| GXEC-D3 | 4 | R | 2 | R | 2 | S |  | S |  | S | 4 | S |  | S |  | S |  | S | 1 | I |  | S |
| GXEC-D4 | 4 | R | 2 | R | 1 | S |  | S | 32 | R | 64 | R | 8 | R | 8 | R | 16 | R | 8 | R |  | S |
| GXEC-D6 | 4 | R | 4 | R | 16 | R | 8 | R | 32 | R | 64 | R | 8 | R | 8 | R | 32 | R | 8 | R |  | S |
| GXEC-E1 | 8 | R | 4 | R | 4 | R |  | S | 32 | R | 64 | R | 8 | R | 8 | R | 16 | R | 1 | I | 8 | I |
| GXEC-E5 | 8 | R | 8 | R | 16 | R | 8 | R | 32 | R | 64 | R | 8 | R | 8 | R | 32 | R | 8 | R | 32 | R |
| GXEC-E6 | 8 | R | 8 | R | 16 | R | 8 | R | 32 | R | 64 | R | 8 | R | 8 | R | 32 | R | 8 | R | 16 | R |
| GXEC-F2 | 8 | R | 4 | R | 2 | S |  | S | 8 | I | 4 | S |  | S |  | S |  | S | 1 | I |  | S |
| GXEC-F9 | 8 | R | 4 | R | 2 | S |  | S | 32 | R | 64 | R | 8 | R | 8 | R | 32 | R | 8 | R | 4 | S |
| GXEC-G8 | 8 | R | 2 | R | 8 | R |  | S | 8 | I | 4 | S |  | S |  | S |  | S | 1 | I |  | S |
| GXEC-H1 | 8 | R | 4 | R | 2 | S |  | S | 32 | R | 64 | R | 8 | R | 8 | R | 8 | I | 8 | R | 8 | I |
| GXEC-H2 | 8 | R | 8 | R | 2 | S | 8 | R | 32 | R | 64 | R | 8 | R | 8 | R | 32 | R | 8 | R | 8 | I |
| GXEC-H10 | 8 | R | 8 | R | 16 | R | 8 | R | 32 | R | 64 | R | 8 | R | 8 | R | 32 | R | 8 | R | 8 | I |
| GXEC-I5 | 8 | R | 8 | R | 16 | R | 8 | R | 32 | R | 64 | R | 8 | R | 8 | R | 32 | R | 8 | R | 16 | R |
| GXEC-I6 | 4 | R | 4 | R | 16 | R |  | S | 32 | R | 64 | R | 8 | R | 8 | R | 4 | S | 8 | R | 16 | R |
| GXEC-J8 | 4 | R | 2 | R | 8 | R | 8 | R | 16 | R | 64 | R | 8 | R | 8 | R | 32 | R | 8 | R |  | S |
| GXEC-K2 | 8 | R | 8 | R | 2 | S |  | S | 32 | R | 16 | I | 2 | I | 0.5 | S |  | S | 1 | I |  | S |

**Table 4** MICs of antimicrobial resistance test (two)

| Strains | MICs（mg/L） | | | | | | | | | | | | | | | | | | | | | |
| --- | --- | --- | --- | --- | --- | --- | --- | --- | --- | --- | --- | --- | --- | --- | --- | --- | --- | --- | --- | --- | --- | --- |
|  | GEN | | AMK | | TET | | DOX | | AZM | | CHL | | SDI | | SXT | | FEP | | CIP | | FOS | |
| GXEC-A1 | 32 | R |  | S | 32 | R | 16 | R | 64 | R | 64 | R | 256 | I |  | S | 4 | R | 256 | R | 32 | R |
| GXEC-A3 | 32 | R | 128 | R | 32 | R | 16 | R | 64 | R | 64 | R | 512 | R |  | S | 4 | R | 256 | R | 32 | R |
| GXEC-A6 | 32 | R |  | S | 32 | R | 16 | R | 32 | R | 32 | R | 256 | I |  | S | 4 | R | 256 | R | 32 | R |
| GXEC-A7 |  | S |  | S | 32 | R | 16 | R |  | S | 64 | R | 256 | I |  | S |  | S |  | S |  | S |
| GXEC-B2 | 32 | R | 128 | R | 32 | R | 16 | R | 64 | R | 64 | R | 256 | I |  | S | 4 | R | 256 | R | 32 | R |
| GXEC-B4 |  | S |  | S | 32 | R | 16 | R |  | S | 32 | R | 256 | I |  | S |  | S |  | S |  | S |
| GXEC-B10 | 32 | R | 128 | R | 32 | R | 16 | R | 64 | R | 64 | R | 256 | I |  | S |  | S | 256 | R | 32 | R |
| GXEC-B11 |  | S |  | S | 32 | R | 16 | R | 64 | R | 64 | R | 512 | R |  | S | 4 | R |  | S |  | S |
| GXEC-C4 |  | S |  | S | 32 | R | 8 | I |  | S |  | S | 256 | I |  | S | 4 | R | 256 | R |  | S |
| GXEC-C6 | 32 | R | 128 | R | 32 | R | 16 | R | 64 | R | 64 | R | 512 | R |  | S | 4 | R | 256 | R | 32 | R |
| GXEC-C12 | 16 | R |  | S | 32 | R | 16 | R |  | S | 64 | R | 256 | I |  | S |  | S |  | S | 16 | R |
| GXEC-C13 | 16 | R |  | S | 32 | R | 16 | R |  | S |  | S | 256 | I |  | S |  | S | 256 | R | 16 | R |
| GXEC-C15 |  | S |  | S | 32 | R | 4 | S |  | S |  | S | 256 | I |  | S | 4 | R |  | S |  | S |
| GXEC-C17 | 8 | S |  | S | 32 | R | 16 | R |  | S | 32 | R | 256 | I |  | S | 4 | R | 256 | R | 8 | S |
| GXEC-C18 | 32 | R | 128 | R | 32 | R | 16 | R | 64 | R | 64 | R | 256 | I |  | S |  | S | 256 | R | 32 | R |
| GXEC-D3 | 16 | R |  | S | 32 | R | 4 | S |  | S |  | S | 256 | I |  | S | 4 | R | 256 | R | 16 | R |
| GXEC-D4 |  | S |  | S | 32 | R | 8 | I |  | S | 64 | R | 256 | I |  | S | 4 | R | 256 | R |  | S |
| GXEC-D6 | 32 | R | 128 | R | 32 | R | 8 | I |  | S | 64 | R | 256 | I |  | S |  | S | 256 | R | 32 | R |
| GXEC-E1 | 32 | R | 128 | R | 32 | R | 16 | R | 64 | R | 32 | R | 256 | I |  | S | 4 | R | 256 | R | 32 | R |
| GXEC-E5 | 32 | R | 128 | R | 32 | R | 16 | R | 64 | R | 64 | R | 512 | R |  | S | 4 | R | 256 | R | 32 | R |
| GXEC-E6 | 32 | R | 128 | R | 32 | R | 16 | R | 64 | R | 64 | R | 512 | R |  | S | 4 | R | 256 | R | 32 | R |
| GXEC-F2 |  | S |  | S | 32 | R | 16 | R |  | S | 64 | R | 256 | I |  | S |  | S |  | S |  | S |
| GXEC-F9 | 32 | R | 128 | R | 32 | R | 16 | R | 64 | R | 64 | R | 256 | I |  | S | 4 | R | 256 | R | 32 | R |
| GXEC-G8 |  | S |  | S | 32 | R | 16 | R |  | S | 64 | R | 256 | I |  | S |  | S |  | S |  | S |
| GXEC-H1 | 32 | R | 128 | R | 32 | R | 16 | R | 64 | R | 64 | R | 256 | I |  | S | 4 | R | 256 | R | 32 | R |
| GXEC-H2 | 32 | R | 128 | R | 32 | R | 8 | I | 64 | R | 64 | R | 256 | I |  | S | 4 | R | 256 | R | 32 | R |
| GXEC-H10 | 32 | R | 128 | R | 32 | R | 8 | I | 64 | R | 64 | R | 256 | I |  | S | 4 | R | 256 | R | 32 | R |
| GXEC-I5 | 32 | R | 128 | R | 32 | R | 16 | R | 64 | R | 64 | R | 512 | R |  | S | 4 | R | 256 | R | 32 | R |
| GXEC-I6 | 32 | R | 128 | R | 32 | R | 16 | R | 64 | R | 32 | R | 512 | R |  | S | 4 | R | 256 | R | 32 | R |
| GXEC-J8 | 32 | R | 128 | R | 32 | R | 8 | I |  | S | 64 | R | 256 | I |  | S | 4 | R | 256 | R | 32 | R |
| GXEC-K2 | 32 | R |  | S | 32 | R | 16 | R | 64 | R | 64 | R | 512 | R |  | S |  | S | 256 | R | 32 | R |

**Table 4** MICs of antimicrobial resistance test (three)

| Strains | MICs（mg/L） | | | | | | | | | | | |
| --- | --- | --- | --- | --- | --- | --- | --- | --- | --- | --- | --- | --- |
|  | TZP | | FOX | | AMP | | TGC | | AMC | | SAM | |
| GXEC-A1 |  | S |  | S | 32 | R |  | S |  | S | 16/8 | I |
| GXEC-A3 |  | S |  | S | 32 | R |  | S |  | S | 16/8 | I |
| GXEC-A6 |  | S |  | S | 32 | R |  | S |  | S | 16/8 | I |
| GXEC-A7 |  | S |  | S | 32 | R |  | S |  | S | 8/4 | S |
| GXEC-B2 |  | S |  | S | 32 | R |  | S |  | S | 16/8 | I |
| GXEC-B4 |  | S |  | S | 32 | R |  | S |  | S | 8/4 | S |
| GXEC-B10 |  | S |  | S | 32 | R |  | S |  | S | 16/8 | I |
| GXEC-B11 |  | S |  | S | 32 | R |  | S |  | S | 8/4 | S |
| GXEC-C4 | 64/4 | R |  | S | 32 | R |  | S |  | S | 32/16 | R |
| GXEC-C6 |  | S |  | S | 32 | R |  | S |  | S | 16/8 | I |
| GXEC-C12 |  | S |  | S | 32 | R |  | S |  | S | 16/8 | I |
| GXEC-C13 |  | S |  | S | 32 | R |  | S |  | S | 8/4 | S |
| GXEC-C15 |  | S |  | S | 32 | R |  | S |  | S | 8/4 | S |
| GXEC-C17 |  | S |  | S | 32 | R |  | S |  | S | 16/8 | I |
| GXEC-C18 |  | S |  | S | 32 | R |  | S |  | S | 16/8 | I |
| GXEC-D3 |  | S |  | S | 16 | I |  | S |  | S | 8/4 | S |
| GXEC-D4 |  | S |  | S | 32 | R |  | S |  | S | 8/4 | S |
| GXEC-D6 | 64/4 | R |  | S | 32 | R |  | S | 8/4 | I | 32/16 | R |
| GXEC-E1 |  | S |  | S | 32 | R |  | S |  | S | 8/4 | S |
| GXEC-E5 | 128/7 | R |  | S | 32 | R |  | S | 8/4 | I | 32/16 | R |
| GXEC-E6 | 128/4 | R |  | S | 32 | R |  | S | 8/4 | I | 32/16 | R |
| GXEC-F2 |  | S |  | S | 32 | R |  | S |  | S | 8/4 | S |
| GXEC-F9 |  | S |  | S | 32 | R |  | S |  | S | 16/8 | I |
| GXEC-G8 |  | S |  | S | 32 | R |  | S |  | S | 8/4 | S |
| GXEC-H1 |  | S |  | S | 32 | R |  | S |  | S | 16/8 | I |
| GXEC-H2 | 128/4 | R |  | S | 32 | R |  | S | 8/4 | I | 32/16 | R |
| GXEC-H10 | 128/4 | R |  | S | 32 | R |  | S |  | S | 32/16 | R |
| GXEC-I5 | 128/4 | R |  | S | 32 | R |  | S | 8/4 | I | 32/16 | R |
| GXEC-I6 |  | S |  | S | 32 | R |  | S |  | S | 16/8 | I |
| GXEC-J8 | 128/4 | R |  | S | 32 | R |  | S | 8/4 | I | 32/16 | R |
| GXEC-K2 |  | S |  | S | 32 | R |  | S |  | S | 8/4 | S |

| **Table 5** Information of 116 samples | | | | |
| --- | --- | --- | --- | --- |
| Area | Number of samples | Number of *mcr-1* positive strains | Number and type of farms | Number of pigs |
| Wuming, Nanning | 24 | 9 | 2 swine breeding farms and 11 family farms | 6000 breeding pigs and 8845 fattening pigs |
| Xixiangtang, Nanning | 16 | 6 | 13 family farmers | 10215 fattening pigs |
| Guilin | 24 | 5 | 2 swine breeding farms and 3 family farms | 10000 breeding pigs and 1964 fattening pigs |
| Liuzhou | 10 | 3 | 1 swine breeding farm | 4800 breeding pigs |
| Laibin | 12 | 2 | 1 swine breeding farm | 5000 breeding pigs |
| Hechi | 11 | 2 | 1 swine breeding farm | 5000 breeding pigs |
| Hezhou | 7 | 4 | 4 family farms | 2568 fattening pigs |
| Yulin | 8 | 1 | 4 family farms | 2683 fattening pigs |
| Qinzhou | 4 | 1 | 2 family farms | 1453 fattening pigs |
